# Ticagrelor Prevents Endothelial Cell Apoptosis through the Adenosine Signalling Pathway in the Early Stages of Hypoxia

**DOI:** 10.3390/biom10050740

**Published:** 2020-05-09

**Authors:** Catherine Feliu, Hélène Peyret, Sylvie Brassart-Pasco, Floriane Oszust, Gaël Poitevin, Philippe Nguyen, Hervé Millart, Zoubir Djerada

**Affiliations:** 1 Department of Pharmacology, Hémostase et Remodelage Vasculaire post-Ischémie (HERVI) E.A.3801, SFR CAP-santé, Reims University Hospital, 51, rue Cognacq-Jay, 51095 Reims CEDEX, France; catherine.feliu@univ-reims.fr (C.F.); helene.peyret@univ-reims.fr (H.P.); floriane.oszust@univ-reims.fr (F.O.); herve.millart@univ-reims.fr (H.M.); 2 UMR CNRS/URCA 7369, Matrice Extracellulaire et Dynamique Cellulaire (MEDyC), Reims University Hospital, SFR CAP-santé, 51, rue Cognacq-Jay, 51095 Reims CEDEX, France; sylvie.pasco@univ-reims.fr; 3 Laboratory of Hematology, E.A.3801, SFR CAP-santé, Reims University Hospital, 51, rue Cognacq-Jay, 51095 Reims CEDEX, France; gael.poitevin@univ-reims.fr (G.P.); pnguyen@chu-reims.fr (P.N.)

**Keywords:** ticagrelor, cytoprotective effect, endothelium, extracellular adenosine, adenosine receptors

## Abstract

Background: Several studies have reported the beneficial effects of anti-platelet drugs in cardioprotection against ischaemia–reperfusion injuries. To date, no studies have focused on the indirect cytoprotective effects of ticagrelor via adenosine receptor on the endothelium. Method: By evaluating cell viability and cleaved caspase 3 expression, we validated a model of endothelial cell apoptosis induced by hypoxia. In hypoxic endothelial cells treated with ticagrelor, we quantified the extracellular concentration of adenosine, and then we studied the involvement of adenosine pathways in the cytoprotective effect of ticagrelor. Results: Our results showed that 10 µM ticagrelor induced an anti-apoptotic effect in our model associated with an increase of extracellular adenosine concentration. Similar experiments were conducted with cangrelor but did not demonstrate an anti-apoptotic effect. We also found that A2B and A3 adenosine receptors were involved in the anti-apoptotic effect of ticagrelor in endothelial cells exposed to 2 h of hypoxia stress. Conclusion: we described an endothelial cytoprotective mechanism of ticagrelor against hypoxia stress, independent of blood elements. We highlighted a mechanism triggered mainly by the increased extracellular bioavailability of adenosine, which activates A2B and A3 receptors on the endothelium.

## 1. Introduction

Vascular endothelial lesions are associated with the death of apoptotic cells and dysfunction, leading to the development of atherosclerosis [1,2,3,4]. Endothelial apoptosis plays a critical role in the progress of apoptosis to neighbouring cardiomyocytes and in the development of ischaemic lesions [2,5]. A study describing the time course of myocardial apoptosis during ischaemia/reperfusion reported that endothelial cells were the first to be affected [5]. Endothelial lesions were reported as the initiator of deleterious cascades of organic lesions [2]. Therefore, endothelial cytoprotection was essential to prevent the development of atherosclerosis and to protect the underlying organ. For example, endothelial cytoprotection was necessary to maintain normal cardiac function after transplantation, primarily by controlling the coronary circulation [6]. The authors demonstrated that myocardial and endothelial protection was essential to switch from myocardial cells protection to heart protection. The endothelium has a variety of constitutive and inducible mechanisms to minimize damage and promote repair [3]. Therefore, pharmacological-induced protection against vascular endothelial apoptosis may limit the development of ischaemic lesions of different organs, particularly at the cardiac, renal, and neurological levels [2,3,6]. Several publications have reported the beneficial effects of P2Y12 receptor antagonists (e.g., clopidogrel, prasugrel, cangrelor, and ticagrelor) in cardioprotection against ischaemia–reperfusion injuries [7,8,9,10,11,12,13,14,15,16,17]. First, the beneficial effects of antiplatelet agents have been attributed to a decrease in platelet aggregation and, indirectly, intravascular coagulation, thus preventing coronary thrombotic re-occlusion. A retrospective analysis by Roubille et al. confirmed a direct cardioprotection effect of clopidogrel in ST-elevated myocardial infarction patients [18]. Authors suggested that cangrelor could interact with blood elements, probably platelets, to induce its cardioprotective effects. Indeed, cangrelor did not demonstrate cardioprotective effects in isolated perfused heart models from mice or rabbits [12,13]. Cohen et al. confirmed this hypothesis in thrombocytopenic rats with 30-min/2-h open chest ischaemia/reperfusion injury [19]. More recently, the same team reported that ticagrelor did not induce cardioprotection during ischaemia/reperfusion (I/R) injury in an ex-vivo cardiac model. The actual mechanism by which P2Y12 antagonists trigger protective signalling during cardiac conditioning remains unknown but may involve platelets [20]. Other studies have reported that the cardioprotective effects of ticagrelor, using in vivo ischaemia-reperfusion heart models, were associated with an increase in heart adenosine concentration [17]. Several studies demonstrated that ticagrelor could reduce endothelial cell proliferation [21] and attenuated vascular dysfunction and atherogenesis through the inhibition of inflammatory activation of endothelial cells [22,23]. However, no studies have focused on the cytoprotective effects of ticagrelor on the endothelium during hypoxia. The purpose of this study was to investigate the cytoprotective effects of ticagrelor in an in vitro model of hypoxic endothelial cells in a cell culture environment, independent of the influence of blood compounds such as platelets. We tested the hypothesis of the involvement of adenosine and its receptors in the contribution of beneficial effects. We validated the model with other markers as cell viability, as there was only apoptosis which was significantly expressed; we then focused on an apoptotic model, a model which has not yet been studied at the endothelial level with ticagrelor as a trigger of cytoprotection. We have demonstrated the protective effect of ticagrelor via another marker; in this case, the extracellular concentration of adenosine. We then evaluated the involvement of adenosine receptors and elucidated intracellular signalling with different antagonists and inhibitors as recommended.

## 2. Results

### 2.1. Hypoxia Stress-Induced Overexpression of A2A, A2B Receptors but Not A3 Receptors

**Figure 1:**
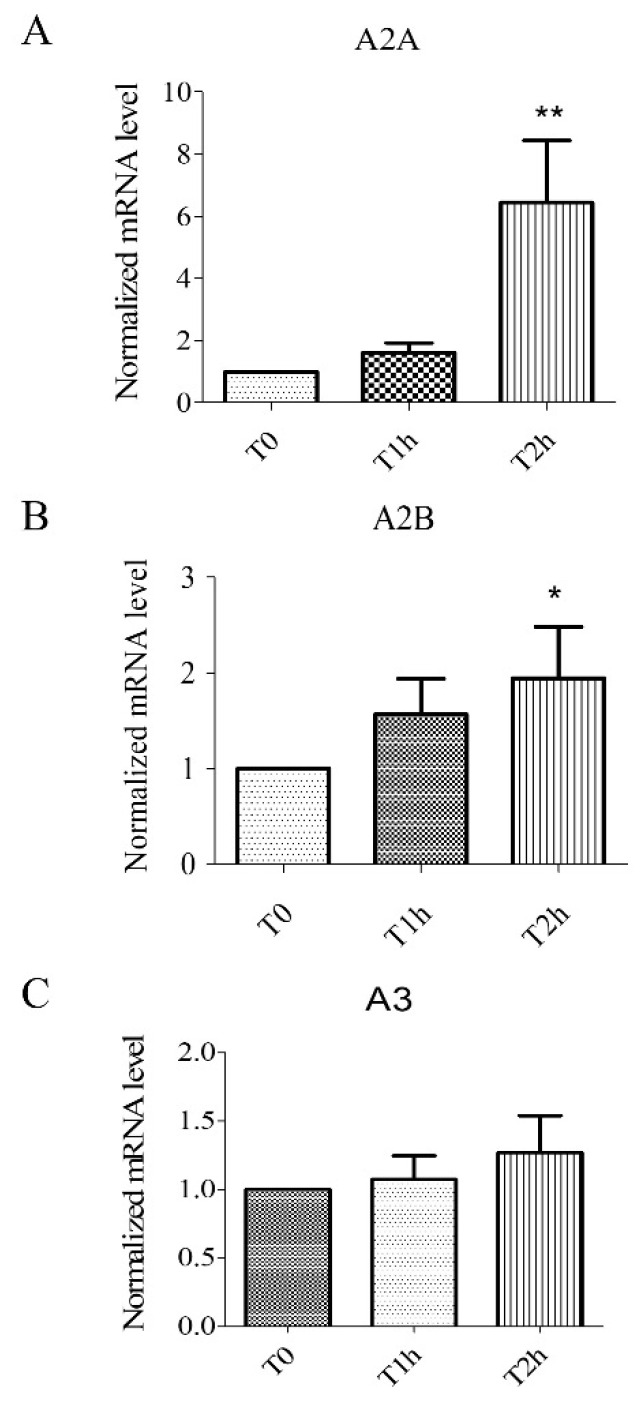
Expression of mRNA for adenosine receptors A2A (**A**), A2B (**B**), and A3 (**C**). Results are expressed with normalized mRNA levels using the following formula: 2^−ΔΔCT^ as a function of time. Overexpression of mRNA for A2A and A2B receptors was observed. Results are expressed as means ± sem (*n* = 6/group). *: *p* < 0.05, **: *p* < 0.01, compared to a normoxic control group.

We studied the expression of adenosine receptors mRNA in human umbilical vein endothelial cells (HUVECs) at different times of hypoxia (T1h and T2h of hypoxia stress). The relative expression to the control group (normoxic control) was estimated as normalized mRNA levels using the following formula: 2^−ΔΔCT^. HUVECs expressed A2A, A2B, and A3 receptors (Figure 1). As previously documented, during hypoxia, over-expression of mRNA was significant (*n* = 6, *p* < 0.05) for A2AAR (T2h: 6.44 ± 1.99) and A2BAR (T2h: 1.94 ± 0.54) (Figure 1). Over-expression of mRNA was also significant (*n* = 6, *p* < 0.05) for A2AAR after 2 h of reoxygenation (T2h-2h: 7.28 ± 2.45, Supplementary data Figure 1).

### 2.2. Effect of Hypoxia-Reoxygenation Stress on Apoptosis Using Different Markers

**Figure 2:**
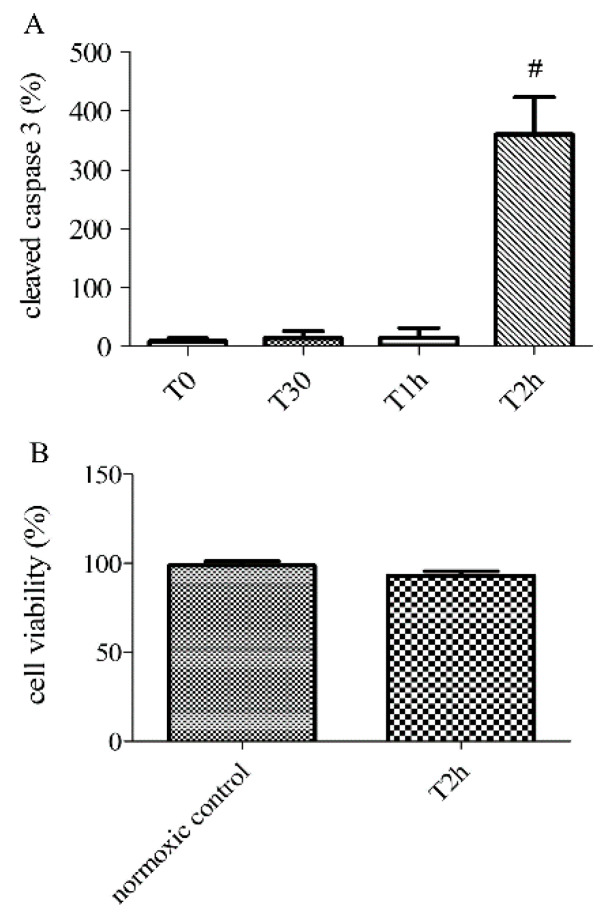
Apoptosis at different time-points of hypoxia (0, 30 min, 1 and 2 h) and cell viability after 0 (normoxic control) or 2 h of hypoxia (T2h), determined by the relative expression of cleaved caspase-3 by immunoblotting (**A**) and PrestoBlue assays (**B**). Results are expressed as means ± sem (*n* = 6/group). #: *p* < 0.05, compared to normoxic control.

HUVECs exposed to 2 h of simulated hypoxia were harvested to determine the expression of cleaved caspase 3. Stress-related to hypoxia resulted in a significant increase in apoptosis. Relative expression of cleaved caspase 3 increased after 2 h of hypoxia: T2h: 360.20 ± 63.50% versus normoxic control group 9.03 ± 4.80% (*n* = 6, *p* < 0.05, Figure 1). Another experiment on HUVECS exposed to 2 h of simulated hypoxia, followed by 2 h of reoxygenation, also induced a significant increase in apoptosis compared to normoxic control: T2h-2h: 400.10 ± 118.10% (Figure 2A). Cell viability was measured 2 h after hypoxia and 2 h after hypoxia followed by 2 h of reoxygenation. The cell viability estimated during experiment also did not change significantly (T2h: 95.42 ± 1.63%, versus normoxic control group 99.30 ± 1.18%, *n* > 6, *p* > 0.05, Figure 2C). The cell viability did not change significantly after 2 h of reoxygenation (T2h-2h 99.33 ± 2.87%, Supplementary data Figure 2). These results confirm that our model can study apoptosis without the confounding effect of cell cytolysis.

### 2.3. Pretreatment with Ticagrelor and Its Effect on Apoptosis

**Figure 3:**
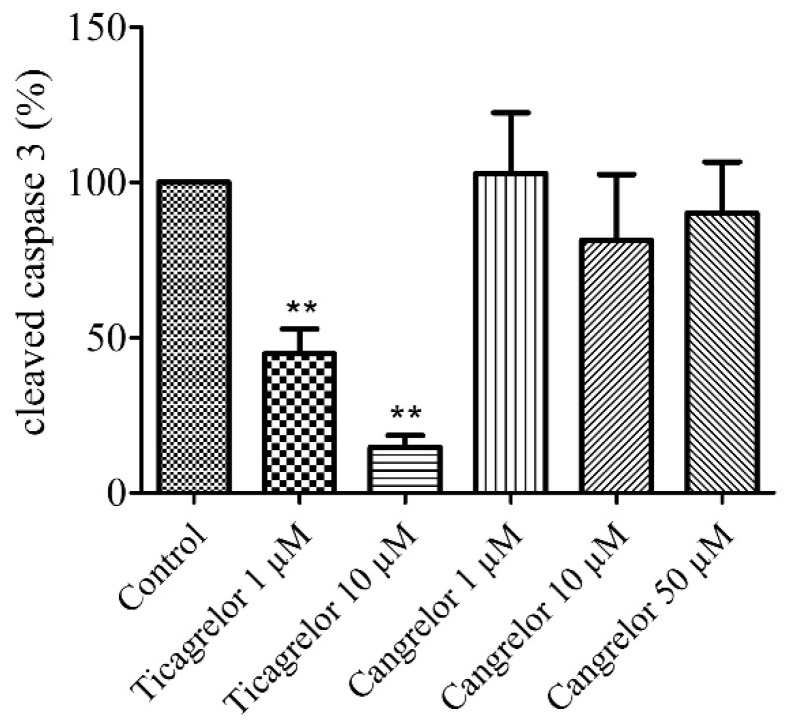
Ticagrelor but not cangrelor induced an anti-apoptotic effect. Cells were treated with ticagrelor 1 µM, 10 µM or with cangrelor 1 µM, 10 µM and 50 µM. Results are expressed as means ± sem (*n* = 6/group) of relative cleaved caspase 3 expression (%) in the human umbilical vein endothelial cells (HUVECs) after 2 h of hypoxia. **: *p* < 0.01 compared to control without any treatment.

Anti-apoptotic effect of 1 µM and 10 µM ticagrelor was assessed in HUVECs after 2 h of hypoxia stress. A significant decrease (*n* = 6, *p* < 0.01) in the relative expression of cleaved caspase 3 was observed in cells treated with 1 µM (44.80 ± 7.92%) and 10 µM ticagrelor (14.67 ± 3.81%), versus control group 100% (untreated group after 2 h of hypoxia stress) (Figure 3, Supplementary data 1).

### 2.4. Pretreatment with Cangrelor and Its Effect on Apoptosis

The anti-apoptotic effect of 1 µM, 10 µM, and 50 µM cangrelor was also assessed in our model after 2 h of hypoxia stress. No significant difference (*n* = 6, *p* > 0.05) in the relative expression of cleaved caspase 3 was observed in cells treated with 1 µM (102.80 ± 19.57%), nor with 10 µM (81.33 ± 21.22%) and 50 µM cangrelor (90.00 ± 16.53%) versus control group 100% (untreated group after 2 h of hypoxia stress) (Figure 3, Supplementary data Figure 3).

### 2.5. Pretreatment with Ticagrelor and Its Effects on Adenosine Extracellular Concentration

**Figure 4:**
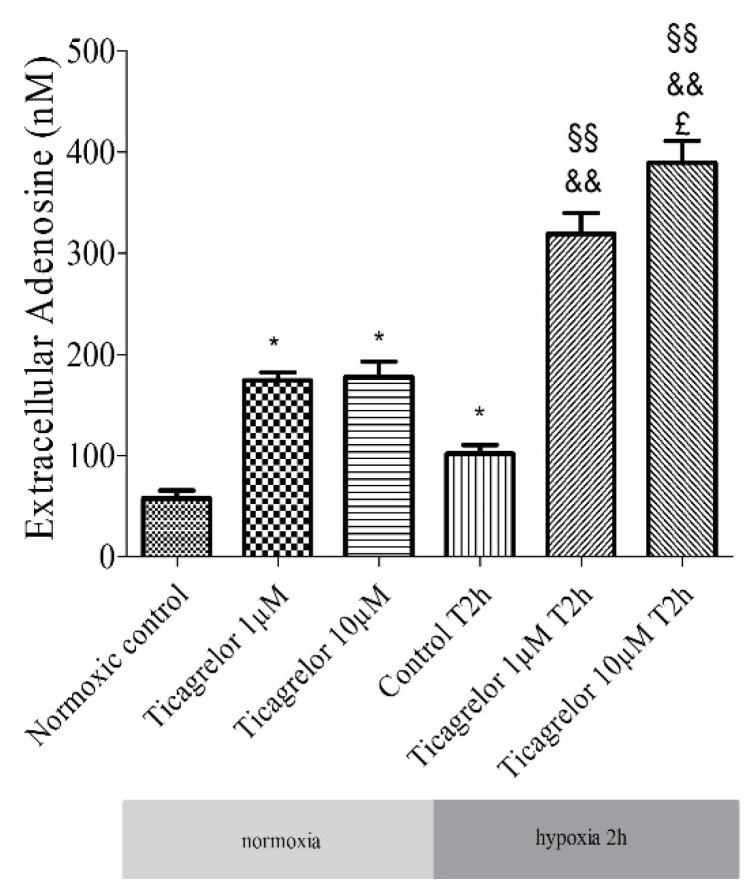
Extracellular adenosine concentration: effect of 1 µM and 10 µM ticagrelor after 2 h of hypoxia stress in HUVECs. Extracellular adenosine concentrations are expressed in nM. Results are expressed as means ± sem (*n* = 6/group). *: *p* < 0.05, compared to the normoxic control group; §§: *p* < 0.01 compared to control T2h, &&: *p* < 0.01 compared to ticagrelor normoxia for each corresponding concentration, £: *p* < 0.05, compared to ticagrelor 1 µM T2h group.

The release of adenosine from HUVECs exposed to 1 µM and 10 µM ticagrelor in the extracellular medium (in 500 µL of supernatant) was studied using liquid chromatography coupled with a high-resolution mass spectrometer (LC-HRMS). Ticagrelor was added 30 min before hypoxia. Extracellular adenosine concentration increased significantly (*n* = 6, *p* < 0.05) in cells treated with ticagrelor (Figure 4, Supplementary data 1). After 30 min of normoxia, the increase was significant (1 µM ticagrelor: 174.02 ± 8.12 nM, 10 µM ticagrelor 177.90 ± 15.03 nM, compared to untreated control group 58.09 ± 7.57 nM). After 2 h of hypoxia stress, adenosine concentrations were 319.30 ± 20.43 nM with 1 µM ticagrelor, 389.62 ± 21.37 nM with 10 µM ticagrelor, and 102.01 ± 8.87 nM in the untreated control group (Figure 4). After 2 h of hypoxia stress followed by 2 h of reoxygenation, adenosine concentrations were 428.72 ± 20.48 nM with 1 µM ticagrelor, 529.34 ± 13.29 nM with 10 µM ticagrelor and 100.40 ± 4.00 nM in untreated control group (Supplementary data Figure 4). The increase in extracellular adenosine concentration was significantly higher in cells treated with 10 µM ticagrelor than in those treated with 1 µM ticagrelor after 2 h of hypoxia and after 2 h of hypoxia followed by 2 h of reoxygenation. The increase was also significantly greater over time regardless of the ticagrelor concentration.

### 2.6. Involvement of Adenosine Receptors in Ticagrelor-Induced-Anti-Apoptotic Effects against Damaging Effects of Hypoxia in Endothelial Cells

**Figure 5:**
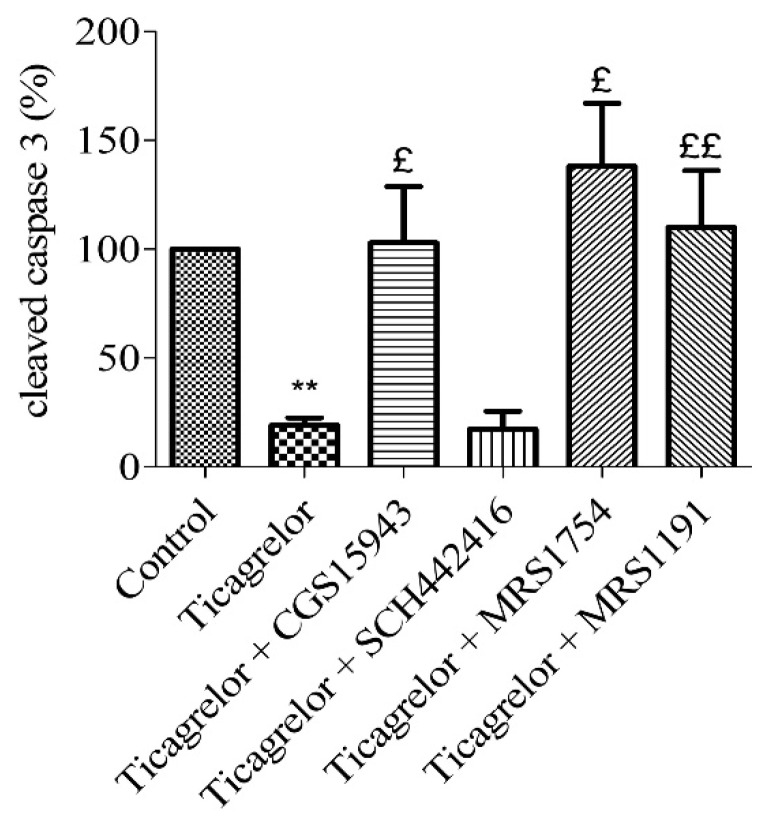
Anti-apoptotic effect of ticagrelor mediated by adenosine receptors. Five minutes prior to 10 µM ticagrelor treatment, HUVECs were treated with different adenosine antagonists. Thirty minutes after ticagrelor treatment, cells were exposed to simulated hypoxia for 2 h. The involvement of adenosine receptors was studied using a non-selective adenosine receptor antagonist (1 µM CGS15943) and selective receptors antagonists of A2A (10 µM SCH442416), A2B (0.1 µM MRS1754) and A3 (10 µM MRS1191) receptors. Data are means ± sem (*n* = 6/group) of relative cleaved caspase 3 expressions (%) in HUVECs after 2 h of hypoxia. £: *p* < 0.05, ££: *p* < 0.01, compared to 10 µM ticagrelor group. **: *p* < 0.01, compared to control without treatment.

The role of adenosine receptors, involved in the anti-apoptotic effect of ticagrelor, was investigated using a panel of selective antagonists (Table 1). Cells were first pretreated with a non-selective adenosine antagonist (1 µM CGS15943) for 5 min and then treated with 10 µM ticagrelor for 30 min prior to hypoxia induction [24] (Figure 5). The anti-apoptotic effect of 10 µM ticagrelor was suppressed by CGS15943 (cleaved caspase 3 related to untreated control group: 103.02 ± 25.69%) compared to cells treated with 10 µM ticagrelor alone (19.14 ± 3.45%, *n* = 6, *p* < 0.05).

To highlight the role of adenosine receptors subtypes in the anti-apoptotic effect of ticagrelor on hypoxic endothelial cells, a pharmacological approach using selective antagonists (Table 1) was used. In the same way as above, HUVECs were treated with 10 µM SCH442416 (a selective A2AAR antagonist) [25], 0.1 µM MRS1754 (selective A2BAR antagonist) [26] or 10 µM MRS1191 (selective A3AR antagonist) [26], five minutes prior ticagrelor 10 µM treatment. The anti-apoptotic effect of 10 µM ticagrelor (19.14 ± 3.45% cleaved caspase-3 compared to control group) was abolished in cells after the blockade of A2BAR (138.00 ± 29.02%) and A3AR (110.00 ± 26.15%) (*n* = 6, *p* < 0.05) (Figure 5). The anti-apoptotic effect of 10 µM ticagrelor was not affected by the blockade of A2AAR (17.43 ± 8.22%).

### 2.7. Anti-Apoptotic Effect of Ticagrelor Involves pi3k, Nos, and Cox Pathways

**Figure 6:**
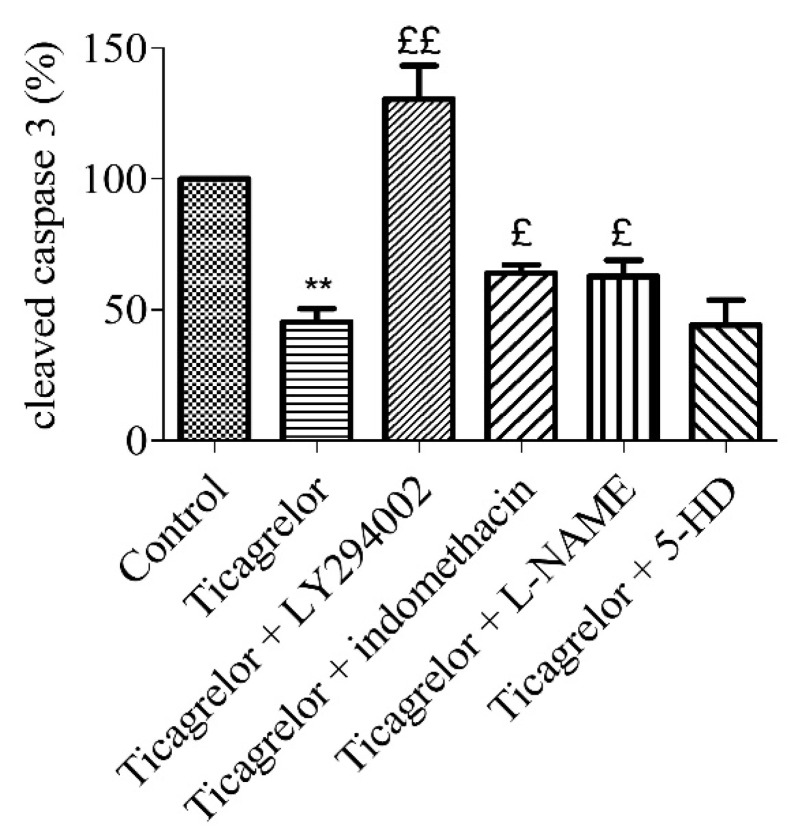
Anti-apoptotic effect of ticagrelor involved PI3K, NOS, and COX. Five minutes prior to 10 µM ticagrelor treatment, HUVECs were pretreated with different selective inhibitors of signalling pathways. Thirty minutes after ticagrelor treatment, cells were exposed to simulated hypoxia for 2 h. Signalling pathways were studied using selective inhibitors pathways of PI3K (10 µM LY294002), mitoKATP (100 µM 5-HD), NOS (10 µM L-NAME) and COX (5 µM, indomethacin). Data are means ± sem (*n* = 6/group) of relative cleaved caspase 3 expression (%) in HUVECs after 2 h of hypoxia. £: *p* < 0.05, ££: *p* < 0.01, compared to 10 µM ticagrelor group. **: *p* < 0.01, compared to control without treatment.

To assess the signalling pathways involved in the ticagrelor-mediated anti-apoptotic effect, five minutes prior to 10 µM ticagrelor treatment, HUVECs were treated with different inhibitors (Table 1): 10 µM LY294002 (PI3K), 100 µM 5-HD (mitoKATP channel), and 10 µM L-NAME (NOS) and 5 µM indomethacin (COX). The anti-apoptotic effect (relative to control) of 10 µM ticagrelor (45.33 ± 5.11%) during hypoxia was limited by blocking PI3K pathway (130.60 ± 12.68%, *p* < 0.05). The anti-apoptotic effect of 10 µM ticagrelor was also significantly and partially abolished by blocking COX (64.00 ± 3.14%), and NOS (62.83 ± 6.05%) (*n* = 6, *p* < 0.05) (Figure 6, Supplementary data 1). This suggests the involvement of PI3K, NOS, and COX in the ticagrelor-mediated anti-apoptotic effect.

## 3. Discussion

In this study, we evaluated the anti-apoptotic effect of ticagrelor against hypoxia stress in HUVECs free from blood elements. We used a model reported to induce apoptosis without affecting cell cytolysis [27,30].

Numerous experimental and clinical studies have suggested that P2Y12 receptor antagonists can directly reduce the size of the myocardial infarction [10,11,12,13,17,26,31,32]. In animal models of acute I/R, ticagrelor, and cangrelor, administered a few minutes before reperfusion, significantly reduced the size of the myocardial infarction [10,12,15,16,17].

Although this protective effect has been well described in in vivo models [10,11], it has yet to be clearly established in isolated models. For example, the cardioprotective effect of cangrelor observed in in vivo models has not been demonstrated in isolated perfused heart models from mice or rabbits [10,13]. To produce its protective effects, this research team has suggested that cangrelor could interact with blood elements, probably platelets. They confirmed this hypothesis in thrombocytopenic rats with open thoracic ischaemia of 30 min, followed by 2 h of reperfusion: cangrelor administered did not reduce the size of the infarction [19]. These results indicated that cangrelor required platelets (or its factors) to trigger its protective effects during I/R injury. Most recently, the cardioprotective effects of ticagrelor were not demonstrated in isolated perfused heart models from mice or rabbits [20]. These studies suggested that, without the presence of blood elements, ticagrelor or cangrelor alone could not induce cardioprotection on an isolated heart [20]. Our present study reports that, in an isolated model of endothelial cells, ticagrelor alone was enough to induce an anti-apoptotic effect against hypoxia stress without the involvement of platelets or other elements of blood. Concerning vascular function, ticagrelor has been reported to improve coronary [33,34] and peripheral arterial function [35]. In a comparative study of different doses of ticagrelor and standard-dose clopidogrel on platelet reactivity and endothelial function in diabetic patients with stable coronary artery disease, He et al. demonstrated that the measurement of flow-mediated dilation (FMD) was higher in ticagrelor groups compared to baseline levels [36].

Many studies have been conducted to understand the mechanisms of ticagrelor protection. Although the association of the increase in adenosine concentration with the protective effects of ticagrelor was reported [15,17], only two in vivo studies have suggested the involvement of adenosine receptors in the cardioprotective effects of ticagrelor. Nevertheless, these studies did not identify the adenosine subtypes receptors involved [16,31]. Experimental and clinical studies have described an increase in extracellular adenosine concentration associated with ticagrelor [14,33,34,37,38,39,40,41,42]. Several studies conducted in dogs [34] or humans [33,35,36] have demonstrated that ticagrelor induced an increase in the effect of exogenous and endogenous adenosine. The mechanism by which ticagrelor induced an extracellular increase in adenosine was not described in this study. Some studies suggested that ticagrelor inhibited ENT-1 transporter, leading to an increase in extracellular adenosine [37,38]. We took this hypothesis as the basis for our model and tested whether ticagrelor induces an increase in extracellular adenosine. Other mechanisms cannot be excluded. Some studies conducted on the platelet membrane have also shown that ticagrelor may induce a reorganization of the membrane and changes the level of expression and localization of membrane lipids and proteins [43,44]. The advantage of our model was to highlight the specific effect of ticagrelor on the endothelium by limiting the confounding factors of integrative models such as the isolated perfused heart model or in vivo models. We reported that 1 µM and 10 µM ticagrelor induced an increase in extracellular adenosine concentration (Figure 4). The simulated (95% confident interval) and pharmacokinetic studies of ticagrelor reported levels in the range of 1.5 to 8 µM [45,46]. Based on the initial anti-apoptotic data (Figure 3), we chose 10 µM of ticagrelor. In our in vitro model of acute exposure, the concentration of 10 µM ticagrelor was selected in order to highlight the potential inhibition effect of the different antagonists used in our study. In our study, after 2 h of hypoxia stress, ticagrelor induced a 3-fold increase in extracellular adenosine concentration compared to the control group, reaching a range between 300 and 400 nM (Figure 4). In a venous blood model incubated with ticagrelor for 1 h, without hypoxia, Löfgren et al. demonstrated that ticagrelor inhibited the clearance of adenosine [47]. In their model, ticagrelor induced a 2-fold increase in the extracellular concentration of adenosine [47]. In our study, adenosine concentrations were considerably lower than in these studies. In addition, our previous research has already demonstrated an anti-apoptotic effect of 10 µM adenosine during hypoxia in an endothelial cell model [48]. Other studies have also reported an increase in the concentration of adenosine (0.3–18 µM) in interstitial fluid during ischaemia/reperfusion in a Langendorff cardiac model without being associated with an increase in apoptosis [49,50,51].

We reported the expression of A2AAR, A2BAR, and A3AR in endothelial cells in accordance with previous studies and literature [48,52,53]. In this study, the use of selective antagonist highlighted the involvement of adenosine receptors in the cytoprotective effect of ticagrelor. We demonstrated that A2BAR and A3AR but not A2AAR were implicated in the anti-apoptotic effect of ticagrelor in endothelial cells.

As described in the literature, our work reported the involvement of A2BAR. Yang et al. studied the cardioprotective effect of another P2Y12 receptor antagonist, cangrelor, during reperfusion in different in vivo animal models [10,11,12]. Concerning A3AR, our result demonstrated its involvement in ticagrelor anti-apoptotic effect in endothelial cells. Compared to other subtypes, A3AR has been identified relatively recently [54,55]. Many studies have documented the protective effects of A3AR against ischaemia, including cardioprotection [54,55,56,57,58,59,60]. A3AR was described in the literature as expressed at the endothelium [52]. Based on our results and the literature, the endothelium may be a potential candidate that could explain the cardioprotective effect of the A3 receptor. A3AR was coupled to Gi proteins and, to some extent, Gq proteins [61]. A3AR-mediated responses triggered phospholipase C stimulation, an increase in inositol triphosphate and intracellular calcium concentrations, and convergence on common effectors, including mKATP [54,55]. A3AR also activated potentially protective signalling pathways, such as ERK1/2 and Akt [54,55,62].

Although widely described in cardioprotection [26,27,62,63], our results did not identify the involvement of A2AAR in ticagrelor-mediated protection. This result was surprising compared to our previous findings, where the involvement of A2AAR was highlighted in the protection of extracellular ATP in the endothelial cells submitted to 2 h of hypoxia stress [48]. The potential differences in adenosine concentrations between the two models could explain this difference. In our previous study, adenosine concentration was tested at 10 µM. In contrast, in this present study, extracellular bioavailability of adenosine was mainly due to the inhibition of its uptake into endothelial cells and was around 0.4 µM after 2 h of hypoxia stress. Another essential point was that, in the previous study, we used 10 µM ATP showing a complementary role for P2Y and adenosine receptors [48]. It was possible that the complementary effect of P2Y and adenosine receptors was expressed at this level of ATP concentration [48]. Another hypothesis is a signalling convergence of A3AR and A2AAR. A2AAR is coupled to Gs proteins subunits, which increases adenylcyclase (AC) activity, while A3AR coupled with Gi protein subunits, decreases AC activity. This hypothesis could be supported by literature reporting an EC50 of 0.73 µM for A2AAR and 0.29 µM for A3AR of adenosine [28]. In addition, as mentioned by Cunha et al., function and pharmacology of A2AAR depended on its localization and interacting partners [29]. Even if we did not highlight its involvement in our study, this does not exclude its role in protection during ischaemia-reperfusion, particularly in cardioprotection. In addition, the ability of SCH442416 to antagonize the A2A receptor may be a limitation in this assay. A significant decrease in the affinity of SCH442416 for the A2A receptor has been demonstrated in medium spiny neurons [64]. This effect was demonstrated at the post-synaptic level, where a heterodimer A2A receptor-D2 receptor was highlighted. We cannot exclude the formation of a heterodimer in our model, which may modify the antagonistic capacity of SCH442416.

As in previous studies, in a complementary experiment, we aimed to test the effect of cangrelor in our model. As ectoenzymes hydrolyze cangrelor, we consider that the effect of cangrelor on P2Y12 receptors is transient. However, at the platelet level, the duration of its effect is estimated to 60 min. This enzymatic hydrolysis can limit the effect of cangrelor via the P2Y12 receptors. Relative expression of P2Y12 receptor was described in the endothelium [65]. The involvement of the P2Y12 receptor in the anti-apoptotic effect of ticagrelor should be limited. In other reported studies, ticagrelor at 3 and 10 µM, and cangrelor at 20, 200, and 400 nM, did not induce a cardioprotective effect in an ex vivo model of Langendorff [13,20]. Furthermore, the same non-involvement of P2Y12 receptor in cardioprotective effects was observed when using cangrelor in an in vivo thrombocytopenic rat model of myocardial I/R [19]. The discrepancy between the protective effect observed in our study and the lack of protection in previous ex vivo studies can be explained by model differences as well as differences in the bioavailability of extracellular or interstitial adenosine [13,20]. As these studies did not quantify the extracellular concentration of adenosine, we were unable to confirm our hypothesis concerning discrepancy.

On the other hand, we can, in part, rule out the hypothesis that blocking P2Y12 ticagrelor triggers an intracellular mechanism responsible for the release of ATP/adenosine. We measured extracellular nucleotides concentrations with and without ticagrelor. As described above, we found that, in normoxic controls, 2 h of hypoxia did not increase ATP, or the sum of ATP, ADP, and AMP (Supplementary data 1). Concentrations were close to the limit of the quantification of our assay method.

The involvement of multiple signalling pathways has been described in ticagrelor-mediated cardioprotection against ischaemia. Our results also confirm the involvement of PI3K pathways, NOS, and COX pathways. Previous studies have reported multiple signalling pathways involved in the cardioprotection of ticagrelor, such as Akt [15,17,31] and ERK1/2 [17]. Studies have also highlighted the roles of AMPK [15,16], COX-2 [16,31], and NOS [17,31]. In addition, Nanhwan et al. [31], in a rat coronary artery ligation model, showed a protective effect of chronic ticagrelor treatment on I/R lesions involving adenosine receptors, COX2, eNOS, and the Akt signalling pathway. Ye et al. [17] reported that ticagrelor induced an increase in adenosine concentrations in the myocardium, leading to Akt phosphorylation, ERK1/2, eNOS, and decreased apoptosis. It also reduced fibrosis and pro-inflammatory mediators. These discrepancies between previous studies and our present result can be explained by differences between in vivo models and in vitro models of isolated heart or in vitro models of cultured HUVECs.

There are some limitations in the present study that may be considered in future projects. First, the monolayer cell culture model we used does not take into account the environment. Other models, such co-cultures with smooth muscle cells, or 3D models could be developed to study the effect of ticagrelor in a more integrative model. Second, the ticagrelor-induced plasma membrane modifications already demonstrated on the platelet membrane could be explored at the endothelial level. The effects of ticagrelor on intracellular and extracellular nucleotide and nucleoside charge as well as the expression of purinergic receptors could also be explored. Finally, in order to evaluate, the percentage of viable (double negative), early apoptotic (A+/PI−), late apoptotic (A+/PI+), and necrotic (A−/PI+) cells, flow cytometry analysis could be performed in addition to caspase-3 experiments and more extensive apoptosis times may be investigated to study late apoptosis.

## 4. Materials and Methods

### 4.1. Cell Culture

As validated and described [27,30], HUVECs were purchased from PromoCell and cultivated according to the manufacturer’s recommendations. Endothelial cell growth medium (PromoCell) containing 2% (*v*/*v*) fetal calf serum (FCS), 0.4% (*v*/*v*) endothelial growth supplement: 0.1 ng/mL human EGF, 1.0 μg/mL hydrocortisone, 1 ng/mL human bFGF, 90 µg/mL heparin, and 1% (*v*/*v*) penicillin/streptomycin (Dutscher, Brumath, France) were used in a completely humid atmosphere at 37 °C and 5% CO_2_. The confluent cells were detached using the PromoCell detachment kit containing 30 mM Hepes, trypsin/EDTA solution (0.04%/0.03%), and trypsin neutralizing solution. The FCS was reduced to 1% 24 h before the experiment. All experiments were performed on subconfluent monolayer endothelial cells (80%) after the third passage.

### 4.2. Experimental Protocol

The cells were placed in a custom hypoxic chamber (Bactron, Sheldon Manufacturing Inc., Cornelius, OR., USA) and exposed to 95% (*v*/*v*) N_2_ and 5% (*v*/*v*) CO_2_ for 2 h at 37 °C. PO_2_ in the hypoxic chamber was reduced to less than 1.5% during the experiments. After 2 h of hypoxia stress, the cells were recovered for analysis or underwent a two-hour reoxygenation phase (5% CO_2_ at 37 °C). At the same time, control cells were cultured under normoxic conditions. During the experiments, cells and media were harvested at different times to quantify nucleotides and to evaluate cleaved caspase 3 by immunoblotting.

Ticagrelor (AstraZeneca, Cambridge, United Kingdom) or Cangrelor (Chiesi Farmaceutici, Parme, Italy) were added 30 min before hypoxia. The concentrations used in our experiments were similar to the concentrations described in humans at the recommended dosages [45,46], or in other studies [66,67], i.e., 1 and 10 µM. Adenosine was purchased from Sigma-Aldrich (St. Louis, MO, USA). Adenosine receptors antagonists CGS15943 (Sigma-Aldrich, St. Louis, MO, USA), SCH442416 (Sigma-Aldrich, St. Louis, MO, USA), MRS1754 (Sigma-Aldrich, St. Louis, MO, USA) and MRS1191 (Sigma-Aldrich, St. Louis, MO, USA) were added just prior to ticagrelor solution. The PI3K-inhibitor LY294002 (Sigma-Aldrich, St. Louis, MO, USA), the mitochondrial KATP (mKATP) channel inhibitor 5-hydroxydecanoate (5-HD, Sigma-Aldrich, St. Louis, MO, USA), the cyclo-oxygenase (COX) inhibitor indomethacin (Sigma-Aldrich, St. Louis, MO, USA), and the nitric oxide synthase (NOS) inhibitor N(ω)-nitro-L-arginine methyl ester (L-NAME, Sigma-Aldrich, St. Louis, MO, USA) were added 5 min before ticagrelor. All these compounds were dissolved in phosphate-buffered saline (PBS) or dimethylsulfoxide (DMSO, Bio Basic Inc, Markham, ON, Canada), according to their solubility. Final DMSO concentration was less than 0.1% in the cell culture medium. Working concentrations of inhibitors and antagonists were the same as previously described (Table 1) [24,25,26,68,69,70,71].

### 4.3. RT-PCR

During each experiment, cells were collected at during normoxia without hypoxia stress (normoxic control), after 2 h of hypoxia (T2h) and after 2 h of reoxygenation (T2h-2h). Total RNA was extracted using RNeasy Mini-KitTM (Qiagen, Courtaboeuf, France). cDNA synthesis was obtained from 1 μg RNA using the iScript cDNA synthesis kit (Biorad, Marnes-la-Coquette, France). Real-time PCR was performed with Sybr Green PCR reagents and analyzed on an ABI Prism 7500 Fast Real- Time PCR System (Applied Biosystems, ThermoFisher Scientific, San Jose, CA, USA). PCR was carried out in duplicate for each sample. EEF2 was used as the housekeeping gene. PCR conditions were as follows: 2 min at 50 °C, 2 min at 95 °C, followed by 40 cycles each consisting of 15 s at 95 °C (denaturation) and 1 min at 60 °C (annealing/extension). The specificity of PCR amplification was checked using a melting curve step from 65 °C up to 95 °C following the final cycle. Results are presented as normalized mRNA levels using the following formula: 2^−ΔΔCT^, according to Livak et al. [64]. mRNA expression of adenosine receptors A2A, A2B and A3 was assayed: forward and reverse oligonucleotide primers are described in Supplementary file 1.

### 4.4. Quantification of Adenosine in Extracellular Medium Liquid Chromatography Coupled with a High-Resolution Mass Spectrometer

Culture medium (50 µL) was collected at different times during the course of each experiment (before hypoxia stress, after 2 h of hypoxia and after 2 h of reoxygenation). In order to inhibit ectonucleotidase activity, 75 µL (60% *v*/*v*) of methanol were added and the extracts were frozen at −80 °C [72,73,74]. Internal standard solution (nicotinamide D4 10 µg.L^−1^) was also added. Mixtures were evaporated under nitrogen at 40 °C and then reconstituted with 100 μL of ice-cold water (LC-MS hypergrade). Liquid chromatography (Hypercarb column 5 µM, 2.1 × 150 mm) coupled with high-resolution mass spectrometer (LC-HRMS) was used for the quantification (ThermoFisher Scientific, San Jose, CA, USA). High-resolution masses for adenosine (C_10_H_13_N_5_O_4_, positive mode ionisation, *m*/*z* 268.10403) and for nicotinamide D4 (C_6_H_2_D_4_N_2_O, positive mode ionisation, *m*/*z* 127.0804) were used for quantification. TraceFinder Forensic 3.3 (ThermoFisher Scientific, San Jose, CA, USA) was used for LC-MS, library management, acquisition and processing (Supplementary data 1). These assays were performed as previously described [51,75,76].

### 4.5. Immunoblotting

The samples for Western blot were collected as follows: after treatment, the cell culture medium was collected, and cells were washed in fresh Phosphate Buffered Saline (PBS). Protein extraction was performed by adding 100 µL of RIPA Buffer (Sigma-Aldrich) supplemented with protease and phosphatase inhibitor cocktails, per well. Samples were sonicated thrice for ten seconds then centrifuged 30 min at 16,000× *g* at 4 °C. Bradford assay was performed to quantify proteins in samples. Equal lysed cellular protein (30 µg) were boiled in sample loading buffer for 5 min before loading on 12.5% SDS–PAGE for electrophoretic separation and subsequently transferred into polyvinylidene difluoride membranes (Immun-Blot PVDF Membrane Bio-Rad, Hercules, CA, USA). After blocking with skimmed milk 5% (*m*/*v*) in TBS-T (Tris-Buffered Saline and TWEEN 20 0.05% (*v*/*v*)), membranes were incubated with the primary monoclonal antibodies anti-cleaved caspase 3 at a dilution of 1:1000 (Cell Signalling, Leiden, The Netherlands) or anti-β-actin at a dilution of 1:5000 (Cell Signalling, Leiden, The Netherlands) overnight at 4 °C. After washing with TBS-T, membranes were incubated with appropriate horseradish peroxidase-labelled secondary antibody (Cell Signalling, Leiden, The Netherlands) for 90 min at room temperature. Immunoreactivity was detected with the Chemiluminescent HRP detection reagent (Millipore, Burlington, MA, USA). Quantification was performed by densitometric analysis using the Quantity One software of ChemiDoc XRS (BioRad). ImageLab was used for reprocessing image and quantification [51]. Densitometric analysis of cleaved caspase 3 band densities were normalized to β-actin.

### 4.6. PrestoBlue Assays

The cell viability reagent PrestoBlue is a membrane-permeable solution based on resazurin. The reducing power of living cells forms the red fluorescent compound resorufin, via the mitochondrial enzymes of viable cells. As a result, this change can be detected by measuring fluorescence and absorbance. During the course of each experiment, PrestoBlue (ThermoFisher Scientific, San Jose, USA) was added to cells 30 min before the studied time (normoxic control, T2h, T2h-2h). After incubation, fluorescence was measured at λ_ex_ 560 nm and λ_em_ 590 nm using the above-mentioned microplate reader. The fluorescence of the T0 group represented 100% cell viability.

### 4.7. Statistics

Statistical analyses were performed using the Prism 4.00 GraphPad Software, San Diego, CA, USA and R 3.1.4 (The R Foundation for Statistical Computing, Vienna, Austria. http://www.r-project.org). All data are expressed as mean ± sem (Standard Error of the Mean). Before applying the parametric unpaired *t*-test, the Gaussian distribution of data was assessed by the Shapiro–Wilk normality test and the Kolmogorov–Smirnov test. Differences between groups for data describing Gaussian distribution were evaluated using one-way analysis of variance (ANOVA) followed with a *t*-test (multiple comparison). When the normality of the distribution was rejected, a non-parametric Kruskal–Wallis test was used for comparisons among groups, followed by post-hoc testing using un-paired Mann–Whitney U tests. For all significant differences concerning primary endpoints, a posteriori powers higher than 80% were checked. Bonferroni correction was applied for multiple comparisons. All *p*-values were two-tailed with statistical significance indicated by a value of *p* < 0.05 [77,78].

## 5. Conclusions

The main conclusions of this study were as follows: (1) ticagrelor induced an anti-apoptotic effect in our model, and (2) ticagrelor induced an increase in extracellular adenosine concentration. This effect was dependent on ticagrelor concentration and duration of exposure. In addition, (3) A2BAR and A3AR receptors were involved in the anti-apoptotic effect of ticagrelor in endothelial cells exposed to 2 h of hypoxia stress.

## Figures and Tables

**Table 1:** Listing of inhibitors and antagonists used in the study. This table described different inhibitors and antagonists used for experiments. Concentrations and reference were also noticed.

Compounds Name	Target	Concentration	Reference
CGS15943	Adenosine receptors antagonist	1 µM	Avanzato et al. [24]
SCH442416	selective receptor antagonist A2A	10 µM	Yu et al. [25]
MRS1754	selective receptor antagonist A2B	0.1 µM	Salie et al. [26]
MRS1191	selective receptor antagonist A3	10 µM	Salie et al. [26]
LY294002	PI3K inhibitor	10 µM	Urban et al. [27]
5-HD	mitoKATP inhibitor	100 µM	Millart et al. [28]
L-NAME	NOS inhibitor	10 µM	Millart et al. [28]
indomethacin	COX inhibitor	5 µM	Alm et al. [29]

## Supplementary Materials

The following are available online at https://www.mdpi.com/2218-273X/10/5/740/s1, Supplementary file 1: Forward and reverse oligonucleotide primers for RT-PCR analysis. Supplementary data 1: data on ATP release, Western blot, and adenosine analysis.
