# Supplementary material for: Ticagrelor Prevents Endothelial Cell Apoptosis through the Adenosine Signalling Pathway in the Early Stages of Hypoxia
Source: Biomolecules. 2020 May 9;10(5):740. doi: 10.3390/biom10050740 (PMC7277469; doi:10.3390/biom10050740)
Supplement: Supplementary file 1 [file biomolecules-10-00740-s001.pdf]

Supplemetary Table 1: List of the primers used in the real time PCR experiements

Gene	Primer sequence
EEF2	Forward: GGCCCTCTTATGATGTATATTTCC
	Reverse: CTGACCTTCAGGCCAGT
A2A	Forward: TCGCCATTGACCGCTAC
	Reverse: CCAGCAGATGGCAATGAT
A2B	Forward: CATCTGTGTCCCGCTCA
	Reverse: CCACCCCAGGAATGGA
A3	Forward: AGAGCTGATTGTAACTGACG
	Reverse: GACAACTTTGGGAGCTT

Supplementary data

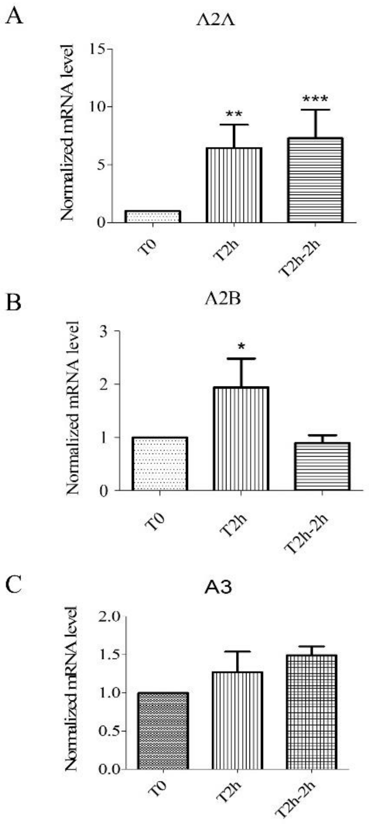

Complement of Figure 1 : mRNA expression after 2 hours of hypoxia and 2 hours of hypoxia followed by 2 hours of reoxygenation for A2A, A2B and A3 receptors.

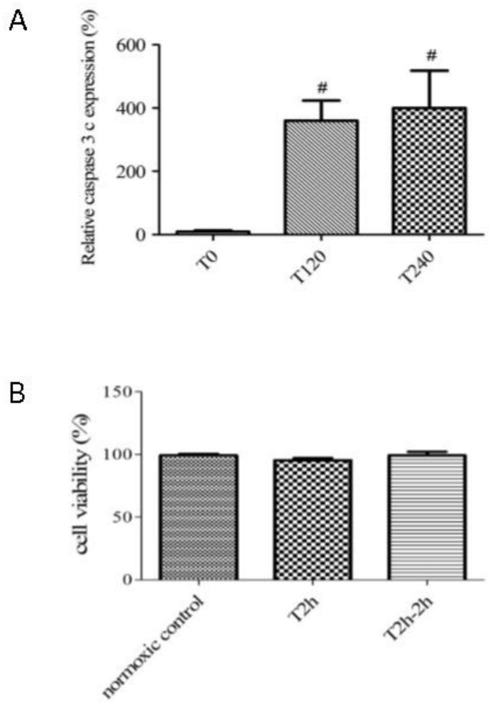

Complement of Figure 2 : Apoptosis and cell viability after 2 hours of hypoxia (T2h) followed by 2 hours of reoxygenation (T2h-2h), determined by the relative expression of cleaved caspase 3 by immunoblotting (A) and PrestoBlue assays (B). Results are expressed as means ± sem (n = 6/group). #: $p < 0.05$, compared to normoxic control.

Extracellular ATP concentrations estimated using LC-HRMS assay.

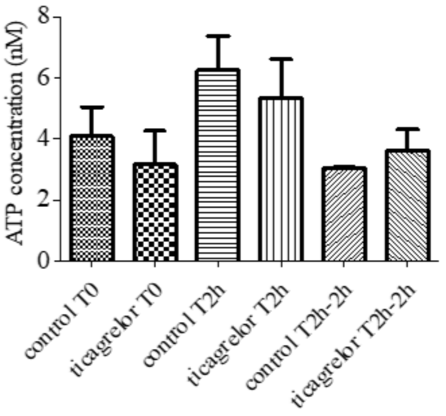

Anova p >0.1 for difference between groups

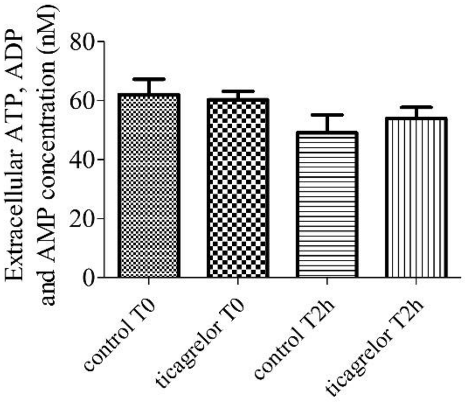

Anova p >0.1 for difference between groups

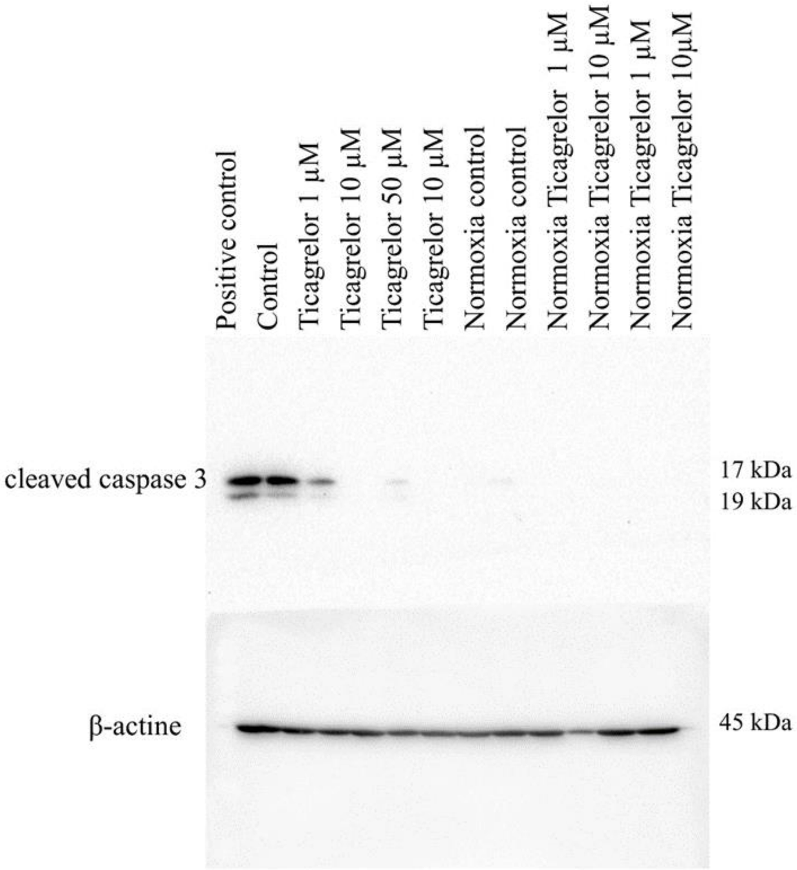

Western blot related to Figure 3

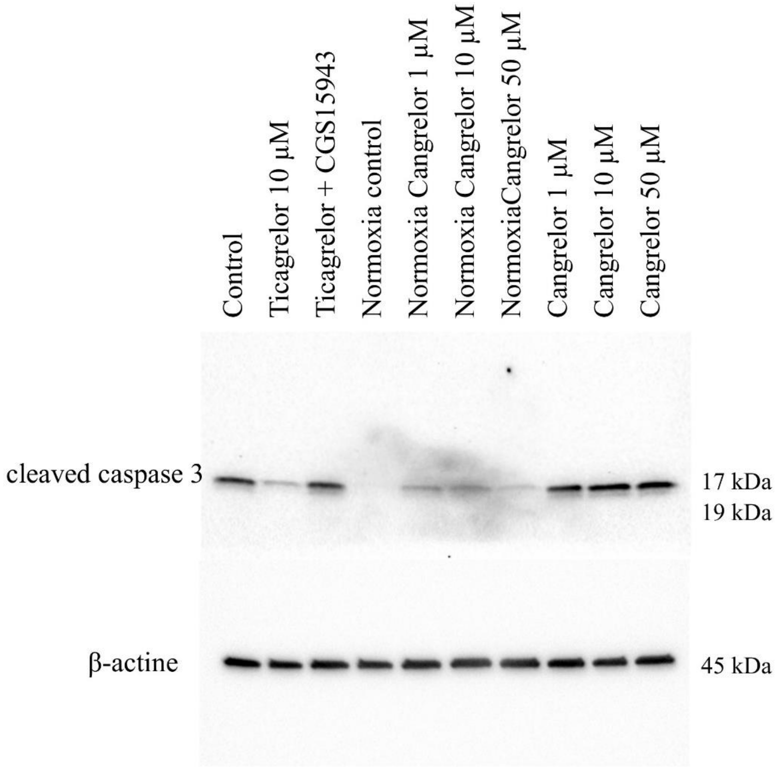

Western blot related to Figure 3

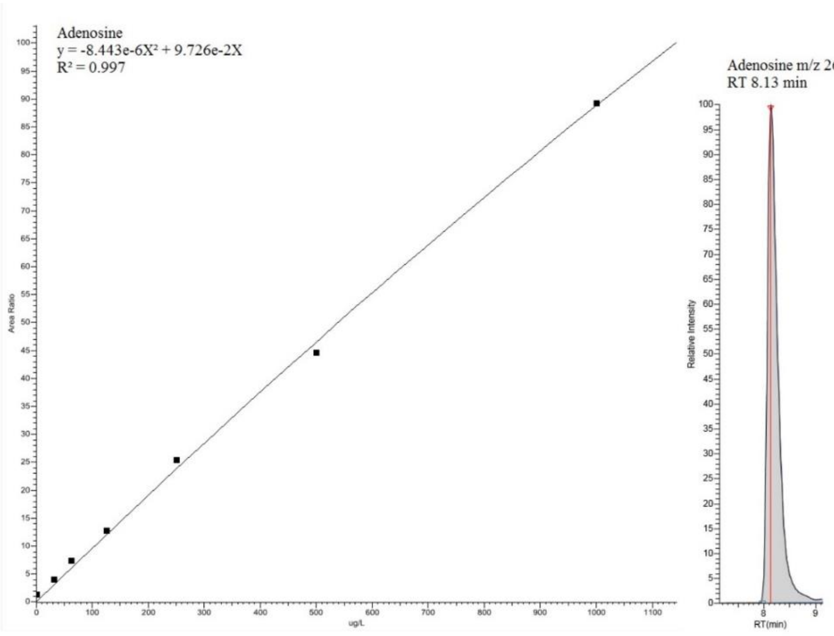

Quantification using liquid chromatography coupled with high resolution mass spectrometry :

Calibration curve of adenosine, coefficient of correlation, retention time, high-resolution masses.

Related to Figure 4

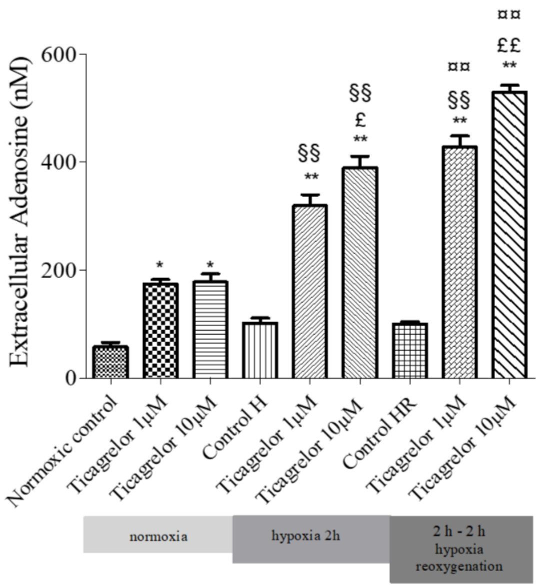

Complement of Figure 4 : Extracellular adenosine concentration: effect of 1 µM and 10 µM ticagrelor during a 2 hours of hypoxia followed by 2 hours of reoxygenation stress in HUVECs. Extracellular adenosine concentrations are expressed in nM. Results are expressed as means ± sem (n = 6/group). *: $p < 0.05$, **: $p < 0.01$, compared to the control groups of the corresponding times; £: $p < 0.05$, ££: $p < 0.01$ compared to one µM ticagrelor for each corresponding times. §§: $p < 0.01$ compared to ticagrelor normoxia for each corresponding concentration. ¤¤: $p < 0.01$ compared to ticagrelor hypoxia for each corresponding concentration.

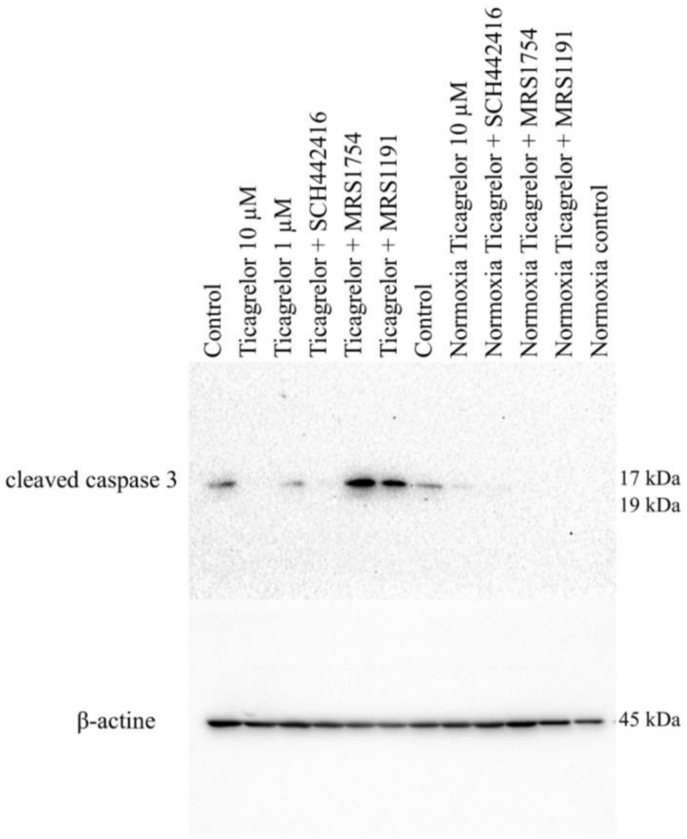

Western blot related to Figure 5

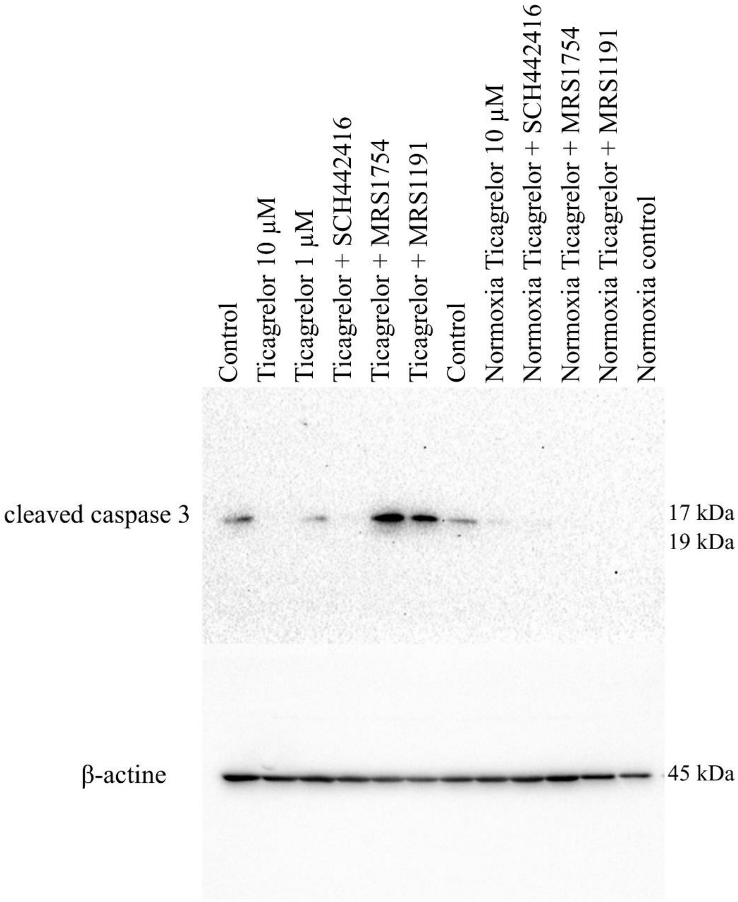

Western blot related to Figure 5
